# Supplementary material for: N-glycolylneuraminic acid serum biomarker levels are elevated in breast cancer patients at all stages of disease
Source: BMC Cancer. 2022 Mar 26;22:334. doi: 10.1186/s12885-022-09428-0 (PMC8962556; doi:10.1186/s12885-022-09428-0)
Supplement: Supplementary file 1 — Additional file 1: Supplementary Information. Supplementary Methods. Figure S1. Glycan array analysis of SubB2M and SubBA12 using a Z-Biotech Neu5Ac/Neu5Gc array. A) Glycan array result of SubB2M and SubBA12 performed using the Z-Biotech Neu5Gc/Neu5Ac N-Glycan Array. Histogram represents the average relative fluorescent units of binding to each of the numbered structures shown in B. For structure ID see http://www.zbiotech.com/neu5gc-xenoantigen-microarray.html and http://nebula.wsimg.com/deda6829116ce09edb871bd7ce7cde6c?AccessKeyId=B5CD53DB37409833427C&disposition=0&alloworigin=1 for further information. Figure S2. Characterization of human CA125 O-glycosylation and bovine Alpha-1-acid glycoprotein (bAGP) by PGC-LC-MS/MS. Annotated Base Peak Chromatogram of the total A) O-glycome released from CA125 and Extracted ion chromatogram of m/z 681.32− (Neu5Gc) and 665.32− (Neu5Ac) and B) N-glycome released from bAGP and Extracted ion chromatogram of m/z 1127.42− (Neu5Gc) and 1111.42− (Neu5Ac). Confirmation of C) Neu5Gc (m/z 681.32−) and Neu5Ac (m/z 665.32−) containing O-glycan structures by MS/MS fragmentation and D) Neu5Gc (m/z 1127.42−) and Neu5Ac (m/z 1111.42−) containing glycan structures by MS/MS fragmentation. Figure S3. A representative Glycoprotein Units (GPUs) standard curve. Bovine AGP (MW = 41–43 kDa; ~ 50%/50% Neu5Ac/Neu5Gc; high total sialic acids) and human CA125 (MW = > 200 kDa, 5–10% Neu5Gc; low total sialic acid) were combined at starting concentrations of 15 μg/ml and 15 units/ml, respectively, in 0.5% normal human serum. This glycoprotein mixture was two-fold serially diluted down to 14.65 ng/ml and 0.0146515 units/ml, respectively, in 0.5% normal human serum. The Response Units (RUs) for each concentration of the standard mixture were determined by subtracting binding due to SubBA12 (flow cell 4) from binding due to SubB2M on flow cell 2 and flow cell 3. RUs obtained for the highest concentration standard was considered 100 GPUs. FC2 = flow cell 2; FC3 [file 12885_2022_9428_MOESM1_ESM.zip › Supplementary information finalR2.pdf]

Supplementary Information

Supplementary Materials and Methods

Development of Glycoprotein Units (GPUs) standard curve for normalization of data from the SubB2M-A_{12}-SPR assays

To generate an internal calibration curve, the Neu5Gc-containing glycoproteins bovine Alpha-1-acid glycoprotein (bAGP) (Sigma-Aldrich, Cat No. G3643) and human cancer antigen 125 (CA125) purified from a human ovarian carcinoma cell line (MyBioSource, San Diego, USA, Cat No. MBS318371) were combined at starting concentrations of 15 µg/ml and 15 units/ml, respectively, in 0.5 % normal human serum (equivalent to 3000 µg/ml bAGP and 3000 units/ml CA125 in 100 % serum). This glycoprotein mixture was two-fold serially diluted down to 14.65 ng/ml and 0.0146515 units/ml, respectively, in 0.5 % normal human serum. This concentration range of glycoprotein standards was run before every set of serum samples analyzed. RUs for each concentration of the GPU standard mixture were determined by subtracting binding due to $SubB_{A12}$ (flow cell 4) from binding due to SubB2M on flow cell 2 and flow cell 3. The RUs obtained for the highest concentration standard was considered 100 GPUs. The resulting standard curve was used to convert SPR RUs, taken at the point of stability in the generated sensorgram, to GPUs. The presence of Neu5Gc on both standard glycoproteins was confirmed by mass spectrometry as described previously (17) and below.

Mass spectrometry glycomic analysis of standard glycoproteins

Glycoproteins (10 µg bAGP and 116.83 units CA125) were immobilised onto PVDF membrane (Millipore) and *N*-glycans released by overnight incubation with 0.5 µl PNGase F (New England BioLabs 500,000 units/ml) in 10 µl water at 37 °C. Released *N*-glycans were reduced to alditols with 0.5 M NaBH4 in 50 mM KOH for 3 h at 50 °C. The reduction was quenched with 1 µl glacial acetic acid desalted using AG50W-X8 cation exchange resin.

O-glycans were released from PNGase F treated proteins by reductive β-elimination. PVDF spots were incubated in 20 µL of 0.5 M $NaBH_4$ in 50 mM KOH at 50 °C for 16 hours and desalted as described for the *N*-glycans.

PGC-LC-ESI-MS *N*-glycans were analysed using a Hypercarb PGC column (3 µm, 100 mm × 180 µm, Thermo Scientific). *N*-glycans were separated over a 90 min and *O*-glycans over a 60 min gradient of 1–90 % of acetonitrile in 10 mM ammonium bicarbonate (vol/vol) at a flow rate of 1 µL/min using a Dionex ultimate HPLC (Thermo Scientific) interfaced with an amaZon Speed ESI-IT mass spectrometer (Bruker Bruker Daltonics, Germany).

The MS spectra were acquired in negative ion mode over a mass range of 450 to 2200 m/z. The following MS settings were used: drying gas temperature: 180 °C, drying gas flow: 5 L/min, nebulizer gas: 9 psi, capillary 3400 V. Ions were detected in ion charge control (ICC) (target: 50,000 ions) with an accumulation time of 200 ms. Induced collision was performed at 35 % normalised collision energy and an isolation window of 4 m/z). Instrument control, data acquisition and processing were performed with Bruker DataAnalysis software version 4.2 (Bruker Daltonics, Germany).

Supplementary Figures

Figure S1. Glycan array analysis of SubB2M and $SubB_{A12}$ using a Z-Biotech Neu5Ac/Neu5Gc array. A) Glycan array result of SubB2M and $SubB_{A12}$ performed using the Z-Biotech Neu5Gc/Neu5Ac N-Glycan Array. Histogram represents the average relative fluorescent units of binding to each of the numbered structures shown in **B**. For structure ID see http://www.zbiotech.com/neu5gc-xenoantigen-microarray.html and http://nebula.wsimg.com/deda6829116ce09edb871bd7ce7cde6c?AccessKeyId=B5CD53DB37409833427C&disposition=0&alloworigin=1 for further information.

Figure S2. Characterization of human CA125 *O*-glycosylation and bovine Alpha-1-acid glycoprotein (bAGP) by PGC-LC-MS/MS. Annotated Base Peak Chromatogram of the total **A)** *O*-glycome released from CA125 and Extracted ion chromatogram of m/z 681.32^- (Neu5Gc) and 665.32^- (Neu5Ac) and **B)** *N*-glycome released from bAGP and Extracted ion chromatogram of m/z 1127.4^{2-} (Neu5Gc) and 1111.4^{2-} (Neu5Ac). Confirmation of **C)** Neu5Gc (m/z 681.32^-) and Neu5Ac (m/z 665.32^-) containing *O*-glycan structures by MS/MS fragmentation and **D**) Neu5Gc (m/z 1127.4^{2-}) and Neu5Ac (m/z 1111.4^{2-}) containing glycan structures by MS/MS fragmentation.

Figure S3. A representative Glycoprotein Units (GPUs) standard curve. Bovine AGP (MW = 41-43 kDa; ~50 %/50 % Neu5Ac/Neu5Gc; high total sialic acids) and human CA125 (MW = >200 kDa, 5-10 % Neu5Gc; low total sialic acid) were combined at starting concentrations of 15 µg/ml and 15 units/ml,

respectively, in 0.5 % normal human serum. This glycoprotein mixture was two-fold serially diluted down to 14.65 ng/ml and 0.0146515 units/ml, respectively, in 0.5 % normal human serum. The Response Units (RUs) for each concentration of the standard mixture were determined by subtracting binding due to $SubB_{A12}$ (flow cell 4) from binding due to SubB2M on flow cell 2 and flow cell 3. RUs obtained for the highest concentration standard was considered 100 GPUs. FC2 = flow cell 2; FC3 = flow cell 3.

Figure S4. ROC curves depicting the ability of serum Neu5Gc levels determined by the optimized SubB2M-A_{12}-SPR assay to distinguish Stage I – IV ovarian cancer patients from cancer-free (normal) individuals. Sensitivity% (true positive rate; ability to detect disease) is plotted against 100 %-specificity% (false positive rate or 100 %-true negative rate; ability to detect lack of disease). ROC analyses were performed with the data shown in Figure 2B using Graphpad Prism 8.0

Figure S5. ROC curves depicting the ability of serum Neu5Gc levels to distinguish Stage I – IV breast cancer patients from normal (cancer-free) individuals. Sensitivity% (true positive rate; ability to detect disease) is plotted against 100 %-specificity% (false positive rate or 100 %-true negative rate; ability to detect lack of disease). ROC analyses were performed with the data shown in Figure 3 using Graphpad Prism 8.0

Figure S6. Serum Neu5Gc levels determined by SubB2M-A_{12}-SPR assay for A) relapse cases and B) remission cases from the Circ.BR cohort.

The mean GPUs from duplicate analyses for each serum sample are shown. Error bars = ± 1 SD from the mean for each group. Two independent assays were performed with both showing the same trends. Results from one assay are shown. Clinical information for each patient is shown in the top right of each plot with treatment history and metastases overlaid. ALND: Axillary lymph node dissection, ILC: Invasive Lobular Carcinoma Mast: mastectomy, SNB: sentinel node biopsy, WLE: wide local excision, XRT: radiation therapy. Detailed information for each patient in the Circ.BR cohort are shown in Supplementary Table 3.

Supplementary Table S4. Optimal cut-off values, sensitivity and specificity for distinguishing Stage I, II, III and IV ovarian cancer patients from normal (cancer-free) individuals using serum Neu5Gc levels determined by optimized SubB2M-SPR assay before and after $SubB_{A12}$ subtraction. Sensitivity and specificity were determined from the Receiver operating characteristic (ROC) curves (**Figure S4**). Optimal cut-off values were selected to give the maximum sum of sensitivity and specificity.

	**Before $SubB_{A12}$ subtraction**	**After $SubB_{A12}$ subtraction**
**Normal vs Stage I**	>9.02 GPUs (91.67 % sensitivity, 100 % specificity)	>6.90 GPUs (100 % sensitivity, 100 % specificity)
**Normal vs Stage II**	>8.83 GPUs (90.91 % sensitivity, 94.45 % specificity)	>6.88 GPUs (100 % sensitivity, 100 % specificity)
**Normal vs Stage III**	>14.50 GPUs (100 % sensitivity, 100 % specificity)	>16.40 GPUs (100 % sensitivity, 100 % specificity)
**Normal vs Stage IV**	>10.49 GPUs (100 % sensitivity, 100 % specificity)	>11.87 GPUs (100 % sensitivity, 100 % specificity)

Supplementary Table S5. Optimal cut-off and area under the curve (AUC) values for distinguishing Stage I, II, III and IV breast cancer patients from normal (cancer-free) individuals using serum Neu5Gc levels. Sensitivity and specificity were determined from the receiver operating characteristic (ROC) curves (**Figure S5**). Optimal cut-off values were selected to give the maximum sum of sensitivity and specificity.

	**Optimal cut-off**	**ROC AUC**
**Normal vs Stage I**	>10.55 GPU (sensitivity = 95.83 %, specificity = 100 %)	0.9583
**Normal vs Stage II**	>10.49 GPU (sensitivity = 100 %, specificity = 100 %)	1.000
**Normal vs Stage III**	>14.54 GPU (sensitivity = 100 %, specificity = 100 %)	1.000
**Normal vs Stage IV**	>19.87GPU (sensitivity = 100%, specificity = 100%)	1.000
