# Supplementary material for: N-glycolylneuraminic acid serum biomarker levels are elevated in breast cancer patients at all stages of disease
Source: BMC Cancer. 2022 Mar 26;22:334. doi: 10.1186/s12885-022-09428-0 (PMC8962556; doi:10.1186/s12885-022-09428-0)

Supplementary Table S1. Supplementary glycan microarray document based on MIRAGE guidelines DOI: 10.1093/glycob/cww118.

Classification	Guidelines
**1. Sample: Glycan Binding Sample**	
Description of Sample	Sample names:<br>*Escherichia coli* SubB2M and $SubB_{A12}$.<br><br>Origin: B subunit pentameric toxins produced as a recombinant protein in *E. coli*.<br><br>Method of preparation:<br>The preparation of SubB2M and $SubB_{A12}$ are explained in the Materials and Methods section.
Sample modifications	SubB2M and $SubB_{A12}$ are hexahistidine-tagged proteins.
Assay protocol	Please see Materials and Methods and appended manufacture's manual.
**2. Glycan Library**	
Glycan description for defined glycans	Arrays used are the Z-Biotech Neu5Gc/Neu5Ac N-Glycan Array. Glycans in this study are listed in Supplementary Figure S1 and are outlined below.
Glycan description for undefined glycans	N/A.
Glycan modifications	N/A
**3. Printing Surface; e.g., Microarray Slide**	
Description of surface	NHS matrix slides
Manufacturer	Schott Nexterion
Custom preparation of surface	N/A.
Non-covalent Immobilisation	N/A.
**4. Arrayer (Printer)**	

Description of Arrayer	See Z-Biotech
Dispensing mechanism	See Z-Biotech
Glycan deposition	See Z-Biotech
Printing conditions	See Z-Biotech
**5. Glycan Microarray with "Map"**	
Array layout	See page 3.
Glycan identification and quality control	Arrays are quality controlled as described on page 6.
**6. Detector and Data Processing**	
Scanning hardware	Innopsys InnoScan 1100AL (Lasers: 488 nM, 532 nM with two filter sets for analysis at 532 and 595 nM), 635 nM) scanner.
Scanner settings	Scanning resolution: 10 µM<br>Laser channel: 532 nM operating 532 nM excitation filter set.<br>PMT: 20 % gain<br>Scan powers: Low laser power.
Image analysis software	Innopsys MAPIX.
Data processing	Data was exported as a CSV file and exported to Microsoft Excel.
**7. Glycan Microarray Data Presentation**	
Data presentation	Data is presented as histograms in Figure S1.
**8. Interpretation and Conclusion from Microarray Data**	
Data interpretation	We only use glycan arrays as a yes/no binding tool. Due to this we look only at binding that is unambiguously above background vs lack of binding above background.
Conclusions	SubB2M is specific for Neu5Gc, $SubB_{A12}$ does not bind to any sialic acid containing glycans.

Print Layout

16-subarray Slide

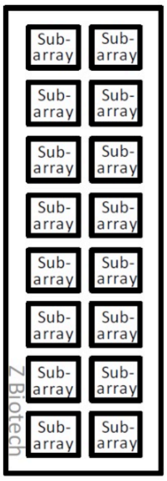

Array Map:

GC-1	GC-1	GC-1	GC-2	GC-2	GC-2	GC-3	GC-3	GC-3	GC-4	GC-4	GC-4	GC-5	GC-5	GC-5	NC1
GC-6	GC-6	GC-6	GC-7	GC-7	GC-7	GC-8	GC-8	GC-8	GC-9	GC-9	GC-9	GC-10	GC-10	GC-10	NC1
GC-11	GC-11	GC-11	GC-12	GC-12	GC-12	GC-13	GC-13	GC-13	GC-14	GC-14	GC-14	GC-15	GC-15	GC-15	NC1
GC-16	GC-16	GC-16	GC-17	GC-17	GC-17	GC-18	GC-18	GC-18	GC-19	GC-19	GC-19	GC-20	GC-20	GC-20	PC1
GC-21	GC-21	GC-21	GC-22	GC-22	GC-22	GC-23	GC-23	GC-23	GC-24	GC-24	GC-24	GC-25	GC-25	GC-25	PC1
GC-26	GC-26	GC-26	GC-27	GC-27	GC-27	GC-28	GC-28	GC-28	GC-29	GC-29	GC-29	GC-30	GC-30	GC-30	PC1
GC-31	GC-31	GC-31	GC-32	GC-32	GC-32	GC-33	GC-33	GC-33	GC-34	GC-34	GC-34	GC-35	GC-35	GC-35	PC2
GC-36	GC-36	GC-36	GC-37	GC-37	GC-37	GC-38	GC-38	GC-38	GC-39	GC-39	GC-39	GC-40	GC-40	GC-40	PC2
AC-1	AC-1	AC-1	AC-2	AC-2	AC-2	AC-3	AC-3	AC-3	AC-4	AC-4	AC-4	AC-5	AC-5	AC-5	PC2
AC-6	AC-6	AC-6	AC-7	AC-7	AC-7	AC-8	AC-8	AC-8	AC-9	AC-9	AC-9	AC-10	AC-10	AC-10	PC3
AC-11	AC-11	AC-11	AC-12	AC-12	AC-12	AC-13	AC-13	AC-13	AC-14	AC-14	AC-14	AC-15	AC-15	AC-15	PC3
AC-16	AC-16	AC-16	AC-17	AC-17	AC-17	AC-18	AC-18	AC-18	AC-19	AC-19	AC-19	AC-20	AC-20	AC-20	PC3
AC-21	AC-21	AC-21	AC-22	AC-22	AC-22	AC-23	AC-23	AC-23	AC-24	AC-24	AC-24	AC-25	AC-25	AC-25	PC4
AC-26	AC-26	AC-26	AC-27	AC-27	AC-27	AC-28	AC-28	AC-28	AC-29	AC-29	AC-29	AC-30	AC-30	AC-30	PC4
AC-31	AC-31	AC-31	AC-32	AC-32	AC-32	AC-33	AC-33	AC-33	AC-34	AC-34	AC-34	AC-35	AC-35	AC-35	PC4
AC-36	AC-36	AC-36	AC-37	AC-37	AC-37	AC-38	AC-38	AC-38	AC-39	AC-39	AC-39	GC-41	GC-41	GC-41	Marker

Glycan list:

N-Glycan Identification List:

Gc Glycan ID	Neu5Gc Glycans	Ac Glycan ID	Neu5Ac Glycans
GC-1	N002G	AC-1	N002
GC-2	N003G	AC-2	N003
GC-3	N005G	AC-3	N005
GC-4	N012G	AC-4	N012
GC-5	N013G	AC-5	N013
GC-6	N015G	AC-6	N015
GC-7	N022G	AC-7	N022
GC-8	N023G	AC-8	N023
GC-9	N025G	AC-9	N025
GC-10	N032G	AC-10	N032
GC-11	N033G	AC-11	N033
GC-12	N042G	AC-12	N042
GC-13	N043G	AC-13	N043
GC-14	N045G	AC-14	N045
GC-15	N052G	AC-15	N052
GC-16	N053G	AC-16	N053
GC-17	N055G	AC-17	N055
GC-18	N112G	AC-18	N112
GC-19	N113G	AC-19	N113
GC-20	N115G	AC-20	N115
GC-21	N122G	AC-21	N122
GC-22	N123G	AC-22	N123
GC-23	N125G	AC-23	N125
GC-24	N133G	AC-24	N133
GC-25	N134G	AC-25	N134
GC-26	N135G	AC-26	N135
GC-27	N144G	AC-27	N144
GC-28	N145G		
GC-29	N155G	AC-29	N155
GC-30	N212G	AC-30	N212
GC-31	N213G	AC-31	N213
GC-32	N215G	AC-32	N215
GC-33	N222G	AC-33	N222
GC-34	N223G	AC-34	N223
GC-35	N225G	AC-35	N225
GC-36	N233G	AC-36	N233
GC-37	N235G		
GC-38	N245G		
GC-39	N255G	AC-39	N255
GC-40	N003G1		
GC-41	N003G2		

Neu5Gc and Neu5Ac N-Glycans

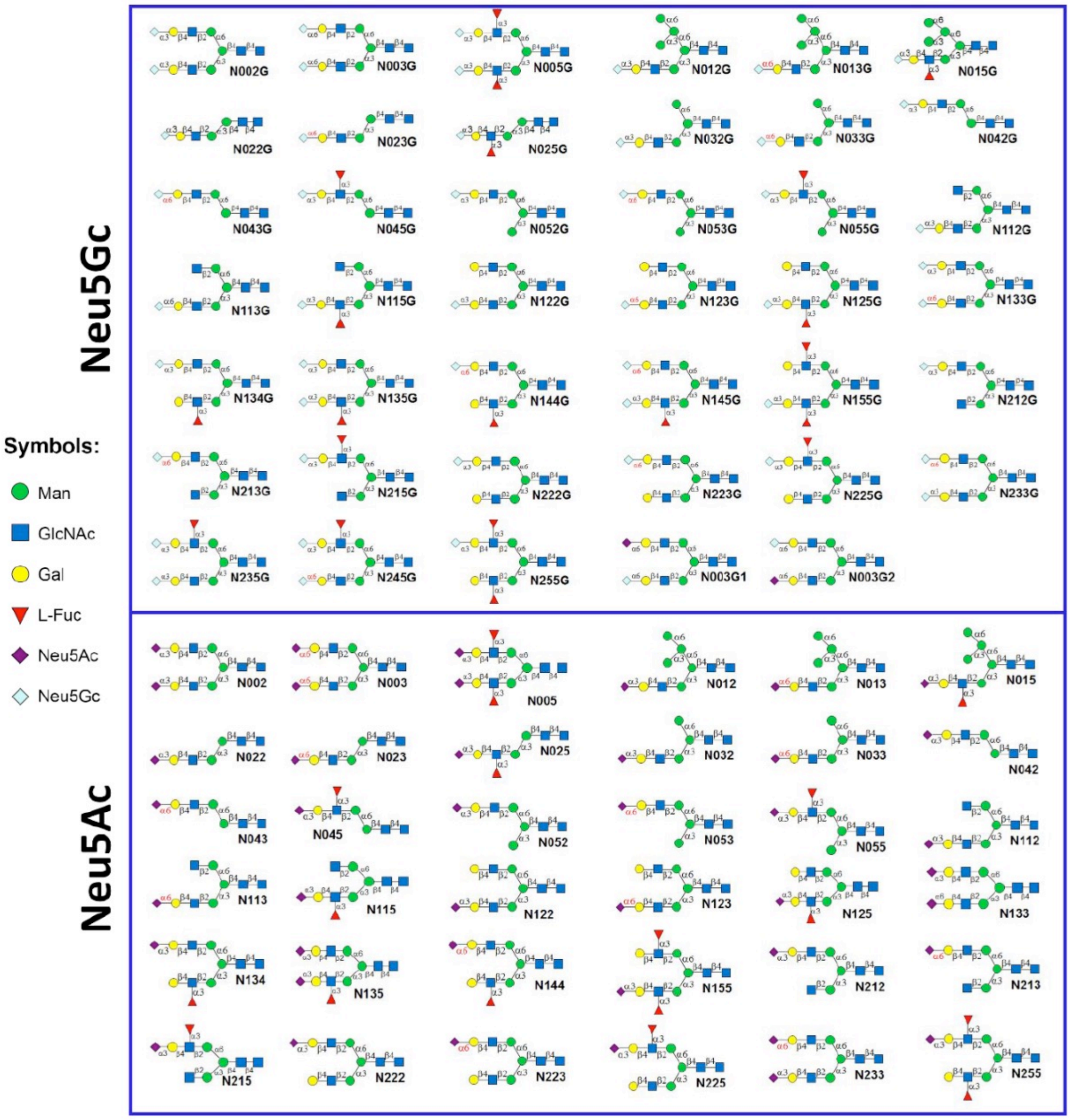

QC

Example 1: Neu5Gc/Neu5Ac array on 8 subarray format. A subarray assayed with a biotinylated SNA target (20 µg/ml), followed by streptavidin-Cy3 (1 µg/ml). The array was scanned with GenePix scanner at 500 PMT and 100% laser power at 532 nm wavelength. The positive control shows binding as expected. N-glycans containing α-2,6 Neu5Gc and α-2,6 Neu5Ac show binding as expected. Analysis of the fluorescence intensity reveals that Neu5Gc-sialylated glycans bind more strongly.

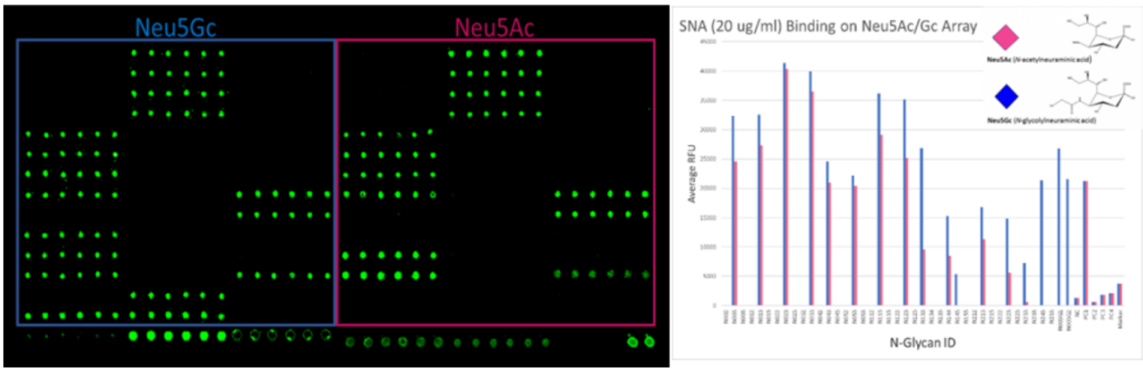

Example 2: Neu5Gc/Neu5Ac array on 8 subarray format. A subarray assayed with a biotinylated WGA target (10 µg/ml), followed by streptavidin-Cy3 (1 µg/ml). The array was scanned with GenePix scanner at 450 PMT and 100% laser power at 532 nm wavelength. The positive control shows binding as expected. Most N-glycans show binding as expected. Analysis of the fluorescence intensity reveals that Neu5Ac-sialylated glycans bind more strongly.

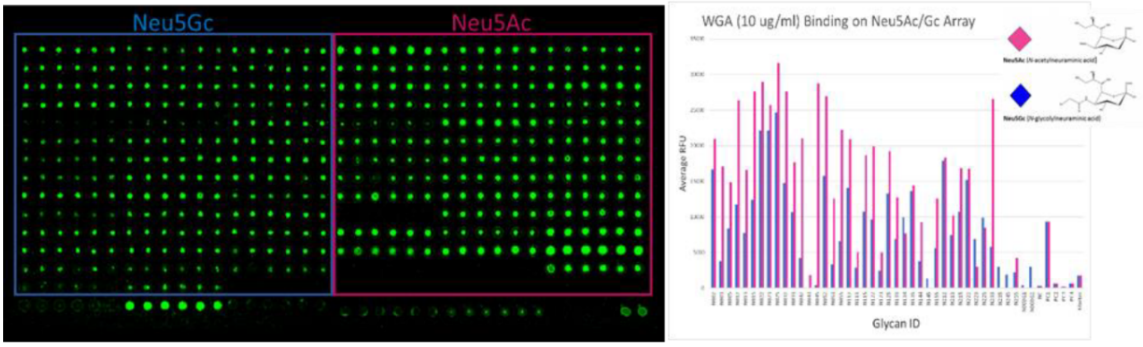

Supplement: Supplementary file 1 — Additional file 1: Supplementary Information. Supplementary Methods. Figure S1. Glycan array analysis of SubB2M and SubBA12 using a Z-Biotech Neu5Ac/Neu5Gc array. A) Glycan array result of SubB2M and SubBA12 performed using the Z-Biotech Neu5Gc/Neu5Ac N-Glycan Array. Histogram represents the average relative fluorescent units of binding to each of the numbered structures shown in B. For structure ID see http://www.zbiotech.com/neu5gc-xenoantigen-microarray.html and http://nebula.wsimg.com/deda6829116ce09edb871bd7ce7cde6c?AccessKeyId=B5CD53DB37409833427C&disposition=0&alloworigin=1 for further information. Figure S2. Characterization of human CA125 O-glycosylation and bovine Alpha-1-acid glycoprotein (bAGP) by PGC-LC-MS/MS. Annotated Base Peak Chromatogram of the total A) O-glycome released from CA125 and Extracted ion chromatogram of m/z 681.32− (Neu5Gc) and 665.32− (Neu5Ac) and B) N-glycome released from bAGP and Extracted ion chromatogram of m/z 1127.42− (Neu5Gc) and 1111.42− (Neu5Ac). Confirmation of C) Neu5Gc (m/z 681.32−) and Neu5Ac (m/z 665.32−) containing O-glycan structures by MS/MS fragmentation and D) Neu5Gc (m/z 1127.42−) and Neu5Ac (m/z 1111.42−) containing glycan structures by MS/MS fragmentation. Figure S3. A representative Glycoprotein Units (GPUs) standard curve. Bovine AGP (MW = 41–43 kDa; ~ 50%/50% Neu5Ac/Neu5Gc; high total sialic acids) and human CA125 (MW = > 200 kDa, 5–10% Neu5Gc; low total sialic acid) were combined at starting concentrations of 15 μg/ml and 15 units/ml, respectively, in 0.5% normal human serum. This glycoprotein mixture was two-fold serially diluted down to 14.65 ng/ml and 0.0146515 units/ml, respectively, in 0.5% normal human serum. The Response Units (RUs) for each concentration of the standard mixture were determined by subtracting binding due to SubBA12 (flow cell 4) from binding due to SubB2M on flow cell 2 and flow cell 3. RUs obtained for the highest concentration standard was considered 100 GPUs. FC2 = flow cell 2; FC3 [file 12885_2022_9428_MOESM1_ESM.zip › Supplementary Table 1R2.pdf]
