# Supplementary material for: N-glycolylneuraminic acid serum biomarker levels are elevated in breast cancer patients at all stages of disease
Source: BMC Cancer. 2022 Mar 26;22:334. doi: 10.1186/s12885-022-09428-0 (PMC8962556; doi:10.1186/s12885-022-09428-0)
Supplement: Supplementary file 1 — Additional file 1: Supplementary Information. Supplementary Methods. Figure S1. Glycan array analysis of SubB2M and SubBA12 using a Z-Biotech Neu5Ac/Neu5Gc array. A) Glycan array result of SubB2M and SubBA12 performed using the Z-Biotech Neu5Gc/Neu5Ac N-Glycan Array. Histogram represents the average relative fluorescent units of binding to each of the numbered structures shown in B. For structure ID see http://www.zbiotech.com/neu5gc-xenoantigen-microarray.html and http://nebula.wsimg.com/deda6829116ce09edb871bd7ce7cde6c?AccessKeyId=B5CD53DB37409833427C&disposition=0&alloworigin=1 for further information. Figure S2. Characterization of human CA125 O-glycosylation and bovine Alpha-1-acid glycoprotein (bAGP) by PGC-LC-MS/MS. Annotated Base Peak Chromatogram of the total A) O-glycome released from CA125 and Extracted ion chromatogram of m/z 681.32− (Neu5Gc) and 665.32− (Neu5Ac) and B) N-glycome released from bAGP and Extracted ion chromatogram of m/z 1127.42− (Neu5Gc) and 1111.42− (Neu5Ac). Confirmation of C) Neu5Gc (m/z 681.32−) and Neu5Ac (m/z 665.32−) containing O-glycan structures by MS/MS fragmentation and D) Neu5Gc (m/z 1127.42−) and Neu5Ac (m/z 1111.42−) containing glycan structures by MS/MS fragmentation. Figure S3. A representative Glycoprotein Units (GPUs) standard curve. Bovine AGP (MW = 41–43 kDa; ~ 50%/50% Neu5Ac/Neu5Gc; high total sialic acids) and human CA125 (MW = > 200 kDa, 5–10% Neu5Gc; low total sialic acid) were combined at starting concentrations of 15 μg/ml and 15 units/ml, respectively, in 0.5% normal human serum. This glycoprotein mixture was two-fold serially diluted down to 14.65 ng/ml and 0.0146515 units/ml, respectively, in 0.5% normal human serum. The Response Units (RUs) for each concentration of the standard mixture were determined by subtracting binding due to SubBA12 (flow cell 4) from binding due to SubB2M on flow cell 2 and flow cell 3. RUs obtained for the highest concentration standard was considered 100 GPUs. FC2 = flow cell 2; FC3 [file 12885_2022_9428_MOESM1_ESM.zip › Supplementary Table 2R2.pdf]

Supplementary Table S2. Details for each of the normal (cancer-free) individuals and breast cancer patients used in this study. Patient details were provided by the Victorian Cancer Biobank with informed written consent from each subject. Abbreviations: ALND: axillary lymph node dissection; DCIS: ductal carcinoma in-situ; G1: Grade 1; G2: Grade 2; G3: Grade 3; LCIS: lobular carcinoma in-situ; LVSI: lymph vascular space invasion; NST: no special type. Information on vital status and/or recurrence was not available from the VCB for all patients.

Specimen no.	Age	Breast cancer stage	Type of breast cancer	Vital status	Date of death	Survival time (years)*	Recurrence	Neu5Gc levels (GPUs)
07AH130	50	N/A	Cancer-free					6.083
08AH714	42	N/A	Cancer-free					6.326
09AH320	47	N/A	Cancer-free					4.972
09AH434	42	N/A	Cancer-free					4.278
09AH794	57	N/A	Cancer-free					10.077
09AH796	52	N/A	Cancer-free					8.653
09AH820	38	N/A	Cancer-free					5.076
12EH0028	53	N/A	Cancer-free					4.694
12EH0114	72	N/A	Cancer-free					5.563
15EH0238	51	N/A	Cancer-free					7.785
15EH0234	79	N/A	Cancer-free					7.125
15EH0228	62	N/A	Cancer-free					6.257
16EH0287	61	N/A	Cancer-free					2.472
16EH0217	76	N/A	Cancer-free					5.806
16EH0396	74	N/A	Cancer-free					3.965
16EH0397	50	N/A	Cancer-free					0.423
17EH0084	54	N/A	Cancer-free					4.556
17EH0260	46	N/A	Cancer-free					7.299
17EH0268	63	N/A	Cancer-free					5.528
17EH0314	46	N/A	Cancer-free					3.792
17EH0349	93	N/A	Cancer-free					3.792
17EH0211	52	N/A	Cancer-free					4.937
02PM1046	80	I	Invasive lobular carcinoma (G2)					20.702

04PM1332	55	I	Invasive ductal carcinoma (G2) with associated DCIS (high grade)	A	N/A			37.856
09PM0065	53	I	Invasive ductal carcinoma (G3) with associated DCIS (high grade)	A	N/A			24.904
15PM0686	38	I	Invasive ductal carcinoma (G2) with associated DCIS (high grade)	A	N/A			11.014
15PM0997	59	I	Invasive mixed micropapillary and ductal/NST carcinoma (G3) and DCIS (high grade)					24.349
11MH0317	43	I	Invasive ductal carcinoma NST (G3)	A	N/A			25.460
12MH0314	44	I	Invasive ductal carcinoma NST (G2) with associated DCIS (high grade)	A	N/A			25.251
11MH0052	68	I	Invasive ductal carcinoma (G2) with associated DCIS (low grade)					16.188
11MH0554	61	I	Invasive ductal carcinoma NST (G2) with associated DCIS (high grade)	A	N/A			22.508
13MH1003	66	I	Invasive ductal carcinoma (G1) with associated DCIS (low grade)					17.299
10RMH737	58	I	Invasive ductal carcinoma NST (G2) with associated DCIS (high grade)					14.799
11MH0567	47	I	Invasive ductal carcinoma with lobular features (G2) with associated DCIS (low grade)					12.369
09RMH083	60	I	Invasive ductal carcinoma NST (G2) with extensive DCIS (high grade)					35.634
10RMH281	54	I	Invasive ductal carcinoma NST (G3)	A	N/A			22.717
10RMH383	77	I	Invasive mucinous adenocarcinoma (G2)					30.391
10RMH514	81	I	Invasive lobular carcinoma NST (G2) with associated LCIS	A	N/A			35.947
12MH0215	35	I	Invasive ductal carcinoma NST (G2)					14.869
12MH0481	56	I	Invasive ductal carcinoma NST (G2) with associated DCIS (high grade)					23.133
12MH1013	65	I	Invasive ductal adenocarcinoma NST (G1) with DCIS (low grade)	A	N/A			-3.674
12MH1291	56	I	Invasive ductal adenocarcinoma NST (G2)	A	N/A			26.849

13MH1223	57	I	Invasive ductal carcinoma NST (G2) with DCIS (intermediate grade)					24.210
14MH0232	51	I	Invasive ductal carcinoma NST (G1)	A	N/A			22.091
14MH0240	43	I	Invasive ductal carcinoma NST (G1) with LCIS (moderate)	A	N/A			28.446
16MH1550	48	I	Lobular carcinoma NST (G3) with associated LCIS	A	N/A			20.772
02PM0520	58	II	Invasive ductal carcinoma NST (G3) with associated DCIS (high grade) & metastatic adenocarcinoma	A	N/A			23.967
04PM0880	45	II	Invasive ductal carcinoma (G3) with associated DCIS (minor high grade) & metastatic carcinoma	A	N/A			17.543
05PM1100	31	II	Mixed invasive ductal carcinoma (NTS and mucinous) (G2) with associated DCIS (high grade) & metastatic tumor of sentinel nodes	A	N/A			11.605
05PM1168	55	II	Invasive ductal carcinoma (G3) with associated DCIS (high grade) & metastatic carcinoma	D	30/05/2008	3		13.410
06PM0542	26	II	Invasive mucinous carcinoma (G1 & G2) with associated DCIS (high grade)					11.257
08PM1308	59	II	Invasive ductal carcinoma NST (G3) & metastatic carcinoma	A	N/A			17.786
08PM1949	49	II	Invasive lobular carcinoma (G2) (post chemotherapy) & metastatic carcinoma	A	N/A			15.772
15PM0781	44	II	Invasive ductal carcinoma NST (G3) & metastatic carcinoma	A	N/A			10.910
14PM0388	67	II	Invasive ductal carcinoma NST (G3) with associated DCIS (high grade)					12.542
15PM1021	53	II	Invasive ductal carcinoma NST (G2) with associated DCIS (intermediate to high grade)	A	N/A			10.945
15PM1119	51	II	Mixed invasive carcinoma (micropapiliary and NST) (G3) with associated DCIS (low and high grade) & metastatic carcinoma	A	N/A			18.619
13MH0429	51	II	Invasive ductal carcinoma (G2) with associated DCIS (intermediate grade) & metastatic carcinoma	D	26/04/2017	4.08		13.063
16MH1592	47	II	Invasive ductal carcinoma NST (G3) with minor DCIS	A	N/A			28.585

01PM0361	75	II	Invasive ductal carcinoma NST (G3) with DCIS (high grade)	D	03/01/2003	1.33		20.286
01PM0632	71	II	Invasive ductal carcinoma NST (G3)	D	06/06/2003	1.75	No	23.411
03PM0734	47	II	Invasive ductal carcinoma NST (G2) & metastatic carcinoma					22.091
05PM2377	49	II	Invasive ductal carcinoma NST (G1) with DCIS (low grade)	A	N/A		Yes	30.738
08PM1773	79	II	Invasive ductal carcinoma NST (G3) with DCIS (high grade)					20.598
08PM1785	54	II	Invasive ductal carcinoma NST (G3) with DCIS (high grade) & metastatic carcinoma	A	N/A			20.771
09PM0040	51	II	Invasive ductal carcinoma NST (G1) with DCIS (high grade)	A	N/A		Yes	16.154
10PM0659	31	II	Invasive ductal carcinoma (G2) with neuroendocrine differentiation					27.092
10PM2156	78	II	Invasive ductal carcinoma NST (G3) with DCIS (high grade)	D	18/03/2017	6.33	No	17.022
12PM0321	46	II	Invasive ductal carcinoma NST (G1) with DCIS (low to intermediate grade) & metastatic carcinoma	A	N/A			15.945
13PM0443	39	II	Invasive ductal (NST) (G3) and lobular carcinoma with DCIS (intermediate to high grade)	D	20/02/2015	1.83	Yes	16.223
01PM0503	49	III	Invasive ductal carcinoma (G3) with associated DCIS (high grade) of right breast	D	09/03/2002	0.42		65.254
03PM0187	57	III	Invasive ductal carcinoma NTS (G3) with associated DCIS (high grade) of left breast with invasion of skeletal muscle and lymphatic channels					30.530
03PM1038	32	III	Invasive ductal carcinoma (G3) with associated DCIS (high grade) of right breast & metastatic carcinoma	D	18/12/2004	1.42		31.016
04PM0931	51	III	Invasive ductal carcinoma NST (G3) with possible DCIS of right breast & metastatic carcinoma, advanced left breast cancer	D	06/01/2005	3		55.879
08PM1472	57	III	Invasive ductal carcinoma (G3) with associated DCIS (high grade) of right breast & metastatic carcinoma					28.967
09PM1880	55	III	Invasive ductal carcinoma (G3) (basal type differentiation) with associated DCIS (high grade) of	A	N/A			41.572

			left breast & metastatic carcinoma					
11PM0575	33	III	Invasive mucinous carcinoma (G1) of left breast & metastatic carcinoma	A	N/A			33.724
11PM1136	42	III	Invasive ductal carcinoma (G3) with associated DCIS (high grade) of right breast & metastatic carcinoma	D	31/08/2013	2.17		37.683
14PM1036	83	III	Invasive ductal carcinoma NST (G3) with associated DCIS (high grade) of right breast & metastatic carcinoma	A	N/A			32.856
15PM0518	53	III	Invasive ductal adenocarcinoma (G3) with associated LCIS (high grade) of left breast & metastatic carcinoma	A	N/A			52.059
15PM0965	56	III	Invasive carcinoma (basal) (G3) with associated DCIS (high grade) of left breast & metastatic carcinoma	A	N/A			73.658
11MH0137	43	III	Invasive ductal carcinoma NST (G3) with associated DCIS (high grade) of right breast & metastatic carcinoma	A	N/A			30.078
09RMH727	45	III	Poorly differentiated invasive ductal carcinoma (G3) & metastatic carcinoma	A	N/A			53.378
10RMH275	69	III	Invasive ductal carcinoma NTS (G1) with DCIS (intermediate to high grade) & metastatic carcinoma					42.857
12MH0211	48	III	Invasive ductal carcinoma (G2) with metastatic ductal carcinoma	A	N/A			41.259
12MH0483	56	III	Micropapillary invasive ductal carcinoma (G3)	A	N/A		Yes	44.142
13MH0077	56	III	Invasive lobular carcinoma (G1) & metastatic lobular carcinoma	A	N/A			53.031
18MH0981	36	III	Invasive ductal carcinoma NTS (G3) with DCIS (low and high grade)	A	N/A			26.363
01PM0213 BLD	66	III	Invasive ductal carcinoma (moderately differentiated) (G2/3) with DICS (intermediate) & metastatic carcinoma					31.120
01PM0463	81	III	Invasive ductal carcinoma (G2) & metastatic carcinoma	D	12/09/2003	2	Yes	38.968
01PM0443	76	III	Invasive ductal carcinoma (G3) with some lobular pattern & metastatic carcinoma	D	20/11/2002	1.17		19.001

02PM1143	39	III	Invasive ductal carcinoma (G2) with associated DCIS (high grade) & metastatic carcinoma	D	05/01/2011	8.08		26.883
03PM0227 BLD	57	III	Invasive ductal carcinoma NTS (G3) with DCIS (high grade)					40.808
08PM2010 BLD	50	III	Invasive ductal carcinoma (G3)	A	N/A		Yes	27.890
17MH0909bl	49	IV	Invasive ductal carcinoma (G3) with associated DCIS (high grade) of left breast with foci of LVSI & metastatic carcinoma	D	06/05/2018	0.83		181.200
17MH0907bl	76	IV	Metastatic breast carcinoma (stable stage IV for 7 years, primary lung cancer)					203.389
15MH1848bl	54	IV	Metastatic adenoid cystic carcinoma of right breast (G3) (also adenoid cystic carcinoma of brain consistent with primary breast cancer)					134.566
15MH1826bl	45	IV	Invasive adenocarcinoma NST (right breast, G3; left breast, G2) with associated DCIS (high grade) & metastatic carcinoma (clavicular head; 6/7 right lymph nodes; 7/7 left lymph nodes), history of Non-Hodgkin lymphoma	A	N/A			138.211
15MH1733bl	27	IV	Invasive ductal carcinoma (G3) with associated DCIS (high grade) of left breast & metastatic carcinoma (4/5 sentinel nodes; 10/11 lymph nodes with extravascular spread)	A	N/A			92.548
13MH1140bl	62	IV	Invasive lobular carcinoma of left breast (G2); Invasive lobular carcinoma (G2) and invasive ductal carcinoma NST (G3) of right breast & metastatic carcinoma (3/18 right axillary lymph nodes)	A	N/A			74.595
13MH0914bl	42	IV	Invasive ductal carcinoma (G3) of left breast; recurrent disease; metastasis to the vertebrae	D	07/12/2013	0.25	Yes	59.108
13MH0767bl	48	IV	Invasive ductal carcinoma (G2) of left breast & metastatic carcinoma (bone metastasis; 1/1 lymph nodes)	D	03/02/2016	2.5		62.962
13MH0529bl	62	IV	Invasive ductal adenocarcinoma (G3) of right breast & metastatic carcinoma (pulmonary metastasis, 2/5 lymph nodes)	D	02/03/2016	2.75		50.774

13MH0217bl	61	IV	Invasive ductal adenocarcinoma (G3) of right breast & metastatic adenocarcinoma (9/12 lymph nodes with extravascular extension; bone metastasis)	A	N/A				36.572
12MH1318	41	IV	Invasive ductal carcinoma (G2) of left breast with associated DCIS (high grade) & metastatic carcinoma (1/13 lymph nodes; pulmonary metastasis)	A	N/A				67.650
12MH0226	66	IV	Invasive ductal carcinoma (G3) of right breast & metastatic carcinoma (19/21 lymph nodes; pulmonary metastasis)	D	12/12/2016	3.75			55.635
02PM0246	73	IV	Longstanding metastatic breast carcinoma (bone, pulmonary and adrenal metastasis)						176.060
02PM0320	64	IV	Invasive primary breast adenocarcinoma (uterus, ovary, fallopian tube and omentum metastasis)						130.884
05PM1349	48	IV	Invasive lobular carcinoma (G2) with minor mucinous component & DCIS (intermediate and high grade) of left breast	D	28/09/2005	0.25			126.856
06PM0159	54	IV	Primary breast carcinoma & metastatic carcinoma (oesophagus)	D	26/12/2006	0.83			88.416
08PM1819	57	IV	Invasive ductal carcinoma of breast (metastasis to left shoulder/chest wall)						77.338
08PM1958	47	IV	Metastatic primary breast carcinoma (left femoral head)						39.002
10PM1401	60	IV	Metastatic primary breast adenocarcinoma (metastasis to brain and spine)						62.476
11PM0548	51	IV	Metastatic primary breast carcinoma (metastasis to brain)						46.607
11PM1336	39	IV	Metastatic breast carcinoma (adenocarcinoma of ovary and fallopian tubes)	A	N/A				29.661
11PM1339	39	IV	Invasive ductal carcinoma NST (G3) with associated DCIS (intermediate to high grade) of both breasts & metastatic breast adenocarcinoma (metastasis to ovaries)	D	29/06/2014	2.83			53.760
13PM0154	72	IV	Metastatic lobular carcinoma (15/18 lymph nodes of left axillary; 10/36 lymph nodes of neck)	A	N/A				35.877

13PM0931	73	IV	Metastatic mucinous carcinoma (metastasis to lung)	A	N/A		Yes	30.148

*From date serum sample was taken to date of death
