# Supplementary material for: N-glycolylneuraminic acid serum biomarker levels are elevated in breast cancer patients at all stages of disease
Source: BMC Cancer. 2022 Mar 26;22:334. doi: 10.1186/s12885-022-09428-0 (PMC8962556; doi:10.1186/s12885-022-09428-0)
Supplement: Supplementary file 1 — Additional file 1: Supplementary Information. Supplementary Methods. Figure S1. Glycan array analysis of SubB2M and SubBA12 using a Z-Biotech Neu5Ac/Neu5Gc array. A) Glycan array result of SubB2M and SubBA12 performed using the Z-Biotech Neu5Gc/Neu5Ac N-Glycan Array. Histogram represents the average relative fluorescent units of binding to each of the numbered structures shown in B. For structure ID see http://www.zbiotech.com/neu5gc-xenoantigen-microarray.html and http://nebula.wsimg.com/deda6829116ce09edb871bd7ce7cde6c?AccessKeyId=B5CD53DB37409833427C&disposition=0&alloworigin=1 for further information. Figure S2. Characterization of human CA125 O-glycosylation and bovine Alpha-1-acid glycoprotein (bAGP) by PGC-LC-MS/MS. Annotated Base Peak Chromatogram of the total A) O-glycome released from CA125 and Extracted ion chromatogram of m/z 681.32− (Neu5Gc) and 665.32− (Neu5Ac) and B) N-glycome released from bAGP and Extracted ion chromatogram of m/z 1127.42− (Neu5Gc) and 1111.42− (Neu5Ac). Confirmation of C) Neu5Gc (m/z 681.32−) and Neu5Ac (m/z 665.32−) containing O-glycan structures by MS/MS fragmentation and D) Neu5Gc (m/z 1127.42−) and Neu5Ac (m/z 1111.42−) containing glycan structures by MS/MS fragmentation. Figure S3. A representative Glycoprotein Units (GPUs) standard curve. Bovine AGP (MW = 41–43 kDa; ~ 50%/50% Neu5Ac/Neu5Gc; high total sialic acids) and human CA125 (MW = > 200 kDa, 5–10% Neu5Gc; low total sialic acid) were combined at starting concentrations of 15 μg/ml and 15 units/ml, respectively, in 0.5% normal human serum. This glycoprotein mixture was two-fold serially diluted down to 14.65 ng/ml and 0.0146515 units/ml, respectively, in 0.5% normal human serum. The Response Units (RUs) for each concentration of the standard mixture were determined by subtracting binding due to SubBA12 (flow cell 4) from binding due to SubB2M on flow cell 2 and flow cell 3. RUs obtained for the highest concentration standard was considered 100 GPUs. FC2 = flow cell 2; FC3 [file 12885_2022_9428_MOESM1_ESM.zip › Supplementary Table 3R2.pdf]

Supplementary Table S3. Details for each of the breast cancer patients from the Circ.BR cohort used in this study. Patient details were provided by the Brisbane Breast Bank with written informed consent from all patients.

Specimen no.	Age	Breast cancer stage	Type of breast cancer	Vital status	Date of death	Survival time (years)*	Recurrence
10-14-183	51	T3N3a	Invasive ductal carcinoma with micropapillary carcinoma	D	25/10/2016	2.2	Yes
10-13-139	74	N/A	Inflammatory breast cancer	D	22/04/2015	4.3	Yes
10-14-093	56	T2N3a	Invasive ductal carcinoma	D	05/08/2016	2.3	Yes
10-14-193	72	T3N2a	Invasive ductal carcinoma	D	04/01/2018	3.4	Yes
10-14-162	51	T2N3a	Invasive ductal carcinoma	D	17/11/2015	1.3	Yes
10-14-092	62	T3Nx	Invasive ductal carcinoma	D	07/12/2015	1.6	Yes
10-14-131	43	T3N3a	Invasive lobular carcinoma	A	N/A		Yes
10-14-062	50	T2N3a	Mixed invasive ductal carcinoma/micropapillary	A	N/A		Yes
10-16-028	69	T2N1	Mixed invasive ductal carcinoma/invasive lobular carcinoma	A	N/A		Yes
10-14-017	45	T3N2	Invasive ductal carcinoma	A	N/A		No
10-14-142	58	T3N1a	Invasive ductal carcinoma	A	N/A		No
10-13-204	39	T3N1MX	Invasive ductal carcinoma	A	N/A		No
10-13-186	53	T1N1c	Invasive ductal carcinoma	A	N/A		No
10-13-157	36	T3N1miM0	Invasive ductal carcinoma	A	N/A		No
10-14-122	37	T2N1a	Mixed metaplastic	A	N/A		No

*Time to death from date of diagnosis
