# Supplementary figures and images for: N-glycolylneuraminic acid serum biomarker levels are elevated in breast cancer patients at all stages of disease
Source: BMC Cancer. 2022 Mar 26;22:334. doi: 10.1186/s12885-022-09428-0 (PMC8962556; doi:10.1186/s12885-022-09428-0)

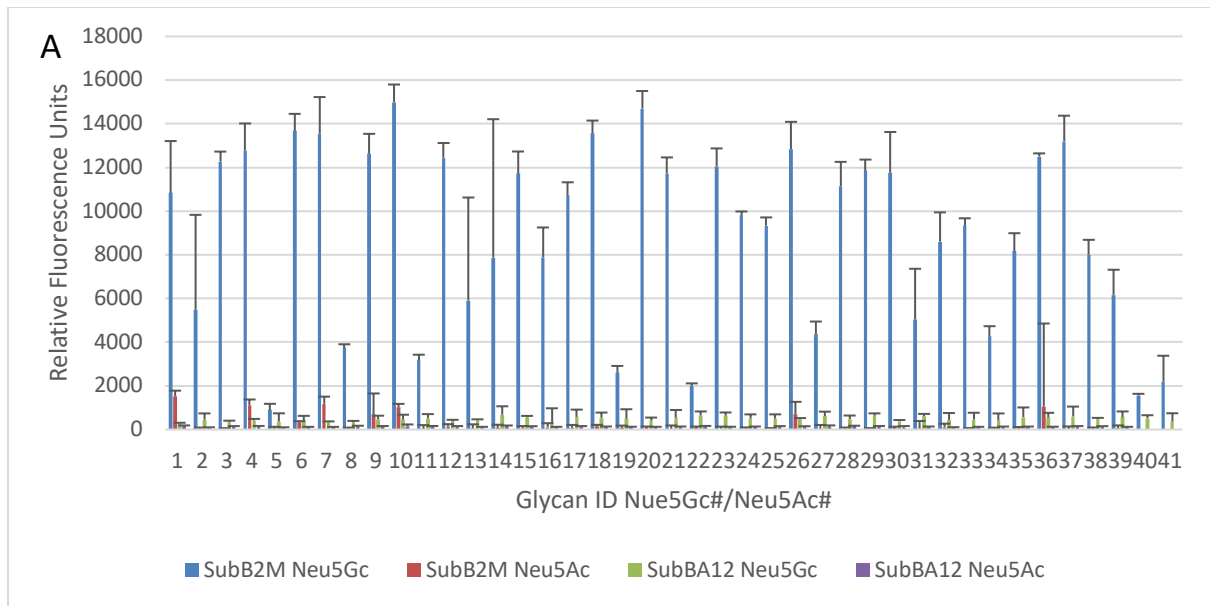

**B Neu5Gc and Neu5Ac N-Glycans**

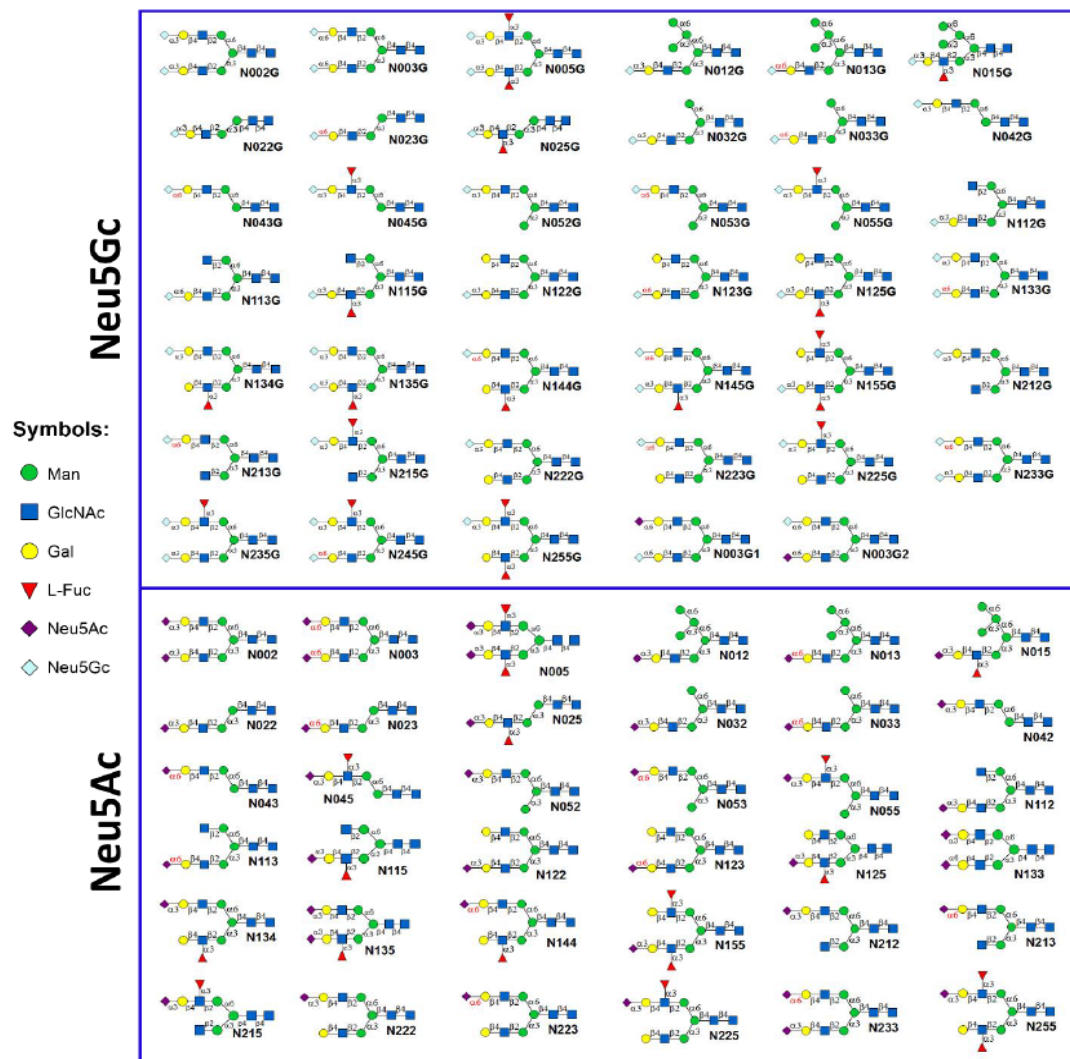

Supplement: Supplementary file 1 — Additional file 1: Supplementary Information. Supplementary Methods. Figure S1. Glycan array analysis of SubB2M and SubBA12 using a Z-Biotech Neu5Ac/Neu5Gc array. A) Glycan array result of SubB2M and SubBA12 performed using the Z-Biotech Neu5Gc/Neu5Ac N-Glycan Array. Histogram represents the average relative fluorescent units of binding to each of the numbered structures shown in B. For structure ID see http://www.zbiotech.com/neu5gc-xenoantigen-microarray.html and http://nebula.wsimg.com/deda6829116ce09edb871bd7ce7cde6c?AccessKeyId=B5CD53DB37409833427C&disposition=0&alloworigin=1 for further information. Figure S2. Characterization of human CA125 O-glycosylation and bovine Alpha-1-acid glycoprotein (bAGP) by PGC-LC-MS/MS. Annotated Base Peak Chromatogram of the total A) O-glycome released from CA125 and Extracted ion chromatogram of m/z 681.32− (Neu5Gc) and 665.32− (Neu5Ac) and B) N-glycome released from bAGP and Extracted ion chromatogram of m/z 1127.42− (Neu5Gc) and 1111.42− (Neu5Ac). Confirmation of C) Neu5Gc (m/z 681.32−) and Neu5Ac (m/z 665.32−) containing O-glycan structures by MS/MS fragmentation and D) Neu5Gc (m/z 1127.42−) and Neu5Ac (m/z 1111.42−) containing glycan structures by MS/MS fragmentation. Figure S3. A representative Glycoprotein Units (GPUs) standard curve. Bovine AGP (MW = 41–43 kDa; ~ 50%/50% Neu5Ac/Neu5Gc; high total sialic acids) and human CA125 (MW = > 200 kDa, 5–10% Neu5Gc; low total sialic acid) were combined at starting concentrations of 15 μg/ml and 15 units/ml, respectively, in 0.5% normal human serum. This glycoprotein mixture was two-fold serially diluted down to 14.65 ng/ml and 0.0146515 units/ml, respectively, in 0.5% normal human serum. The Response Units (RUs) for each concentration of the standard mixture were determined by subtracting binding due to SubBA12 (flow cell 4) from binding due to SubB2M on flow cell 2 and flow cell 3. RUs obtained for the highest concentration standard was considered 100 GPUs. FC2 = flow cell 2; FC3 [file 12885_2022_9428_MOESM1_ESM.zip › Figure S1R2.pdf]

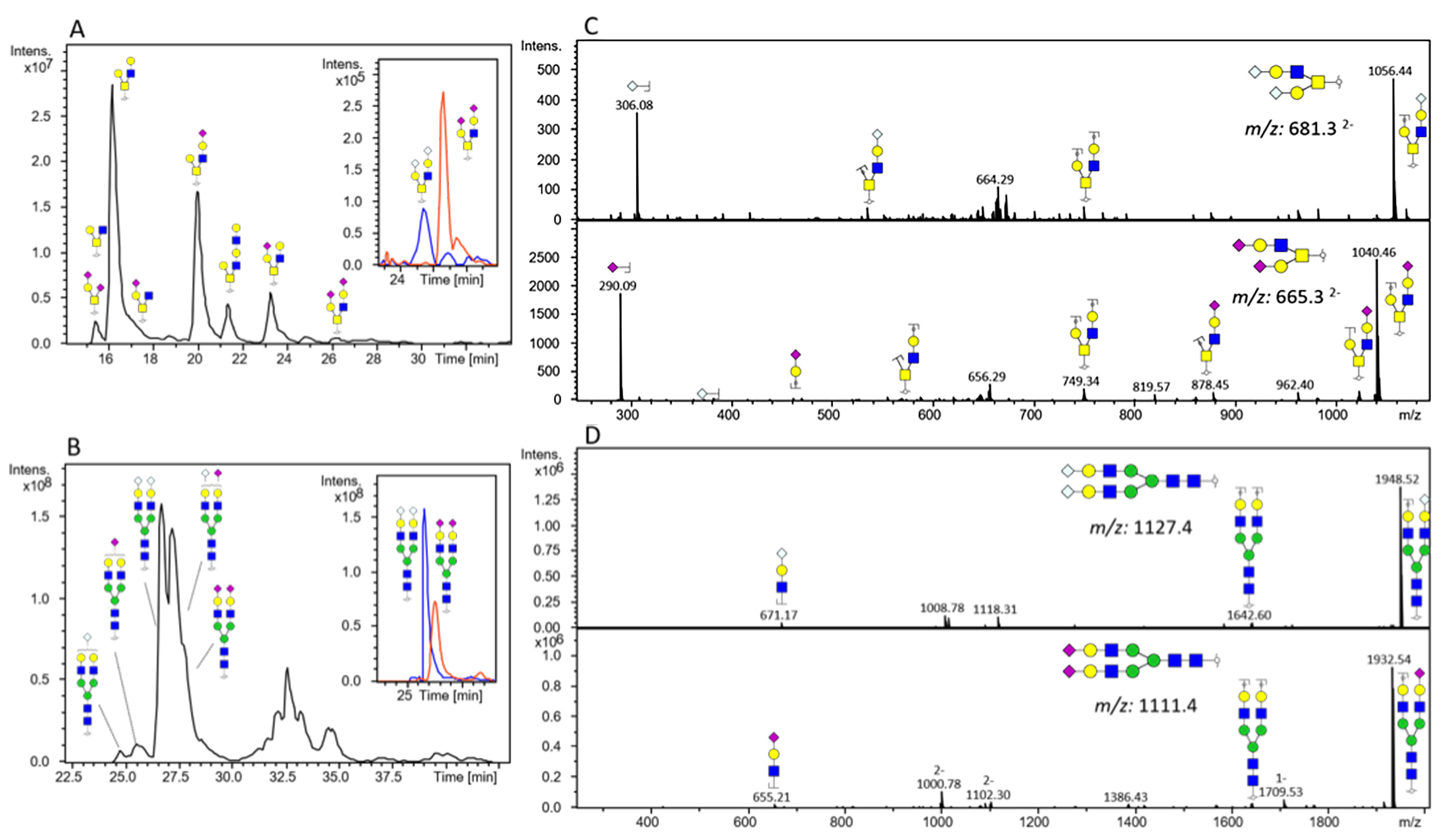

Supplement: Supplementary file 1 — Additional file 1: Supplementary Information. Supplementary Methods. Figure S1. Glycan array analysis of SubB2M and SubBA12 using a Z-Biotech Neu5Ac/Neu5Gc array. A) Glycan array result of SubB2M and SubBA12 performed using the Z-Biotech Neu5Gc/Neu5Ac N-Glycan Array. Histogram represents the average relative fluorescent units of binding to each of the numbered structures shown in B. For structure ID see http://www.zbiotech.com/neu5gc-xenoantigen-microarray.html and http://nebula.wsimg.com/deda6829116ce09edb871bd7ce7cde6c?AccessKeyId=B5CD53DB37409833427C&disposition=0&alloworigin=1 for further information. Figure S2. Characterization of human CA125 O-glycosylation and bovine Alpha-1-acid glycoprotein (bAGP) by PGC-LC-MS/MS. Annotated Base Peak Chromatogram of the total A) O-glycome released from CA125 and Extracted ion chromatogram of m/z 681.32− (Neu5Gc) and 665.32− (Neu5Ac) and B) N-glycome released from bAGP and Extracted ion chromatogram of m/z 1127.42− (Neu5Gc) and 1111.42− (Neu5Ac). Confirmation of C) Neu5Gc (m/z 681.32−) and Neu5Ac (m/z 665.32−) containing O-glycan structures by MS/MS fragmentation and D) Neu5Gc (m/z 1127.42−) and Neu5Ac (m/z 1111.42−) containing glycan structures by MS/MS fragmentation. Figure S3. A representative Glycoprotein Units (GPUs) standard curve. Bovine AGP (MW = 41–43 kDa; ~ 50%/50% Neu5Ac/Neu5Gc; high total sialic acids) and human CA125 (MW = > 200 kDa, 5–10% Neu5Gc; low total sialic acid) were combined at starting concentrations of 15 μg/ml and 15 units/ml, respectively, in 0.5% normal human serum. This glycoprotein mixture was two-fold serially diluted down to 14.65 ng/ml and 0.0146515 units/ml, respectively, in 0.5% normal human serum. The Response Units (RUs) for each concentration of the standard mixture were determined by subtracting binding due to SubBA12 (flow cell 4) from binding due to SubB2M on flow cell 2 and flow cell 3. RUs obtained for the highest concentration standard was considered 100 GPUs. FC2 = flow cell 2; FC3 [file 12885_2022_9428_MOESM1_ESM.zip › Figure S2R2.jpg]

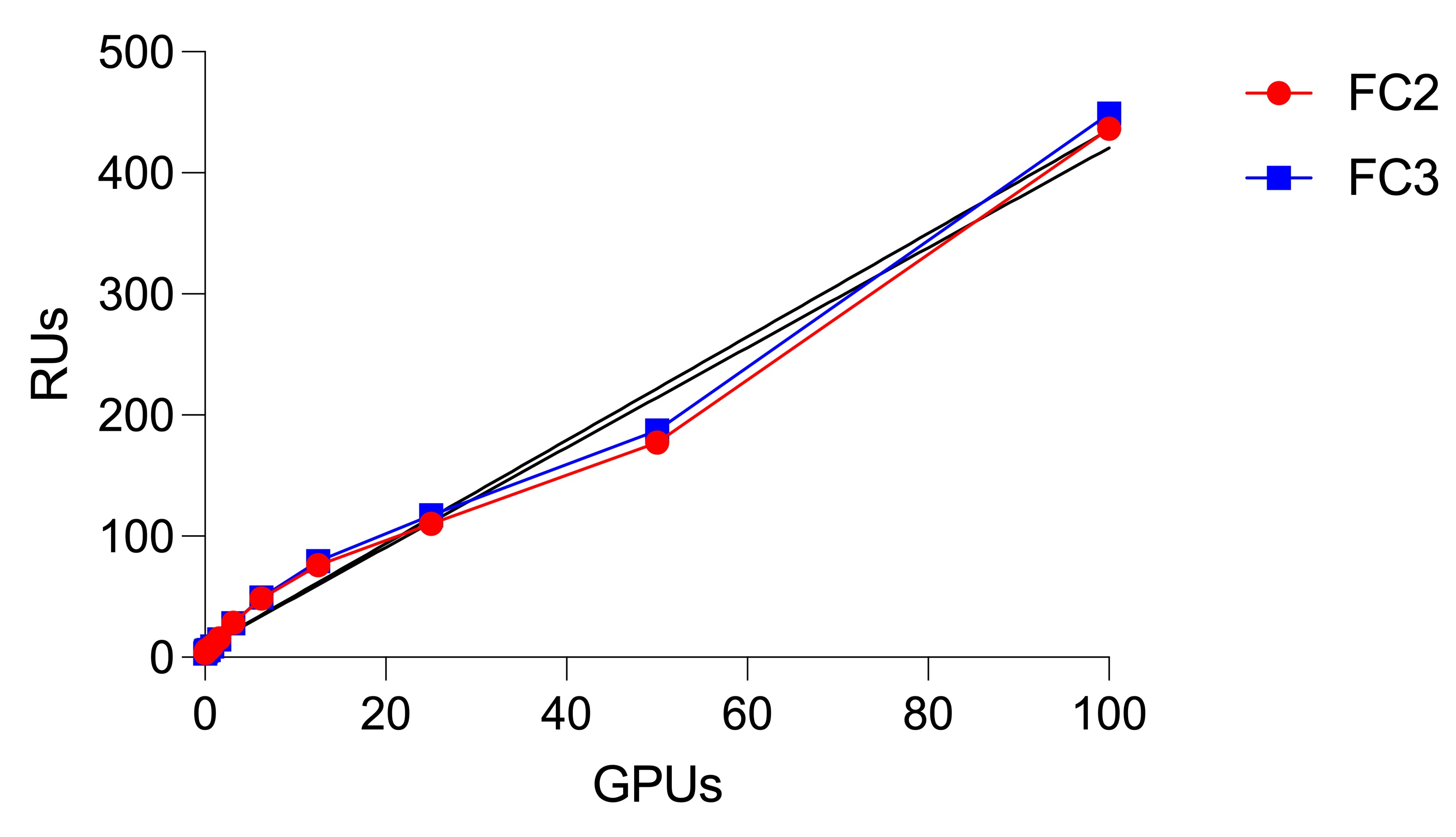

Supplement: Supplementary file 1 — Additional file 1: Supplementary Information. Supplementary Methods. Figure S1. Glycan array analysis of SubB2M and SubBA12 using a Z-Biotech Neu5Ac/Neu5Gc array. A) Glycan array result of SubB2M and SubBA12 performed using the Z-Biotech Neu5Gc/Neu5Ac N-Glycan Array. Histogram represents the average relative fluorescent units of binding to each of the numbered structures shown in B. For structure ID see http://www.zbiotech.com/neu5gc-xenoantigen-microarray.html and http://nebula.wsimg.com/deda6829116ce09edb871bd7ce7cde6c?AccessKeyId=B5CD53DB37409833427C&disposition=0&alloworigin=1 for further information. Figure S2. Characterization of human CA125 O-glycosylation and bovine Alpha-1-acid glycoprotein (bAGP) by PGC-LC-MS/MS. Annotated Base Peak Chromatogram of the total A) O-glycome released from CA125 and Extracted ion chromatogram of m/z 681.32− (Neu5Gc) and 665.32− (Neu5Ac) and B) N-glycome released from bAGP and Extracted ion chromatogram of m/z 1127.42− (Neu5Gc) and 1111.42− (Neu5Ac). Confirmation of C) Neu5Gc (m/z 681.32−) and Neu5Ac (m/z 665.32−) containing O-glycan structures by MS/MS fragmentation and D) Neu5Gc (m/z 1127.42−) and Neu5Ac (m/z 1111.42−) containing glycan structures by MS/MS fragmentation. Figure S3. A representative Glycoprotein Units (GPUs) standard curve. Bovine AGP (MW = 41–43 kDa; ~ 50%/50% Neu5Ac/Neu5Gc; high total sialic acids) and human CA125 (MW = > 200 kDa, 5–10% Neu5Gc; low total sialic acid) were combined at starting concentrations of 15 μg/ml and 15 units/ml, respectively, in 0.5% normal human serum. This glycoprotein mixture was two-fold serially diluted down to 14.65 ng/ml and 0.0146515 units/ml, respectively, in 0.5% normal human serum. The Response Units (RUs) for each concentration of the standard mixture were determined by subtracting binding due to SubBA12 (flow cell 4) from binding due to SubB2M on flow cell 2 and flow cell 3. RUs obtained for the highest concentration standard was considered 100 GPUs. FC2 = flow cell 2; FC3 [file 12885_2022_9428_MOESM1_ESM.zip › Figure S3R2.jpg]

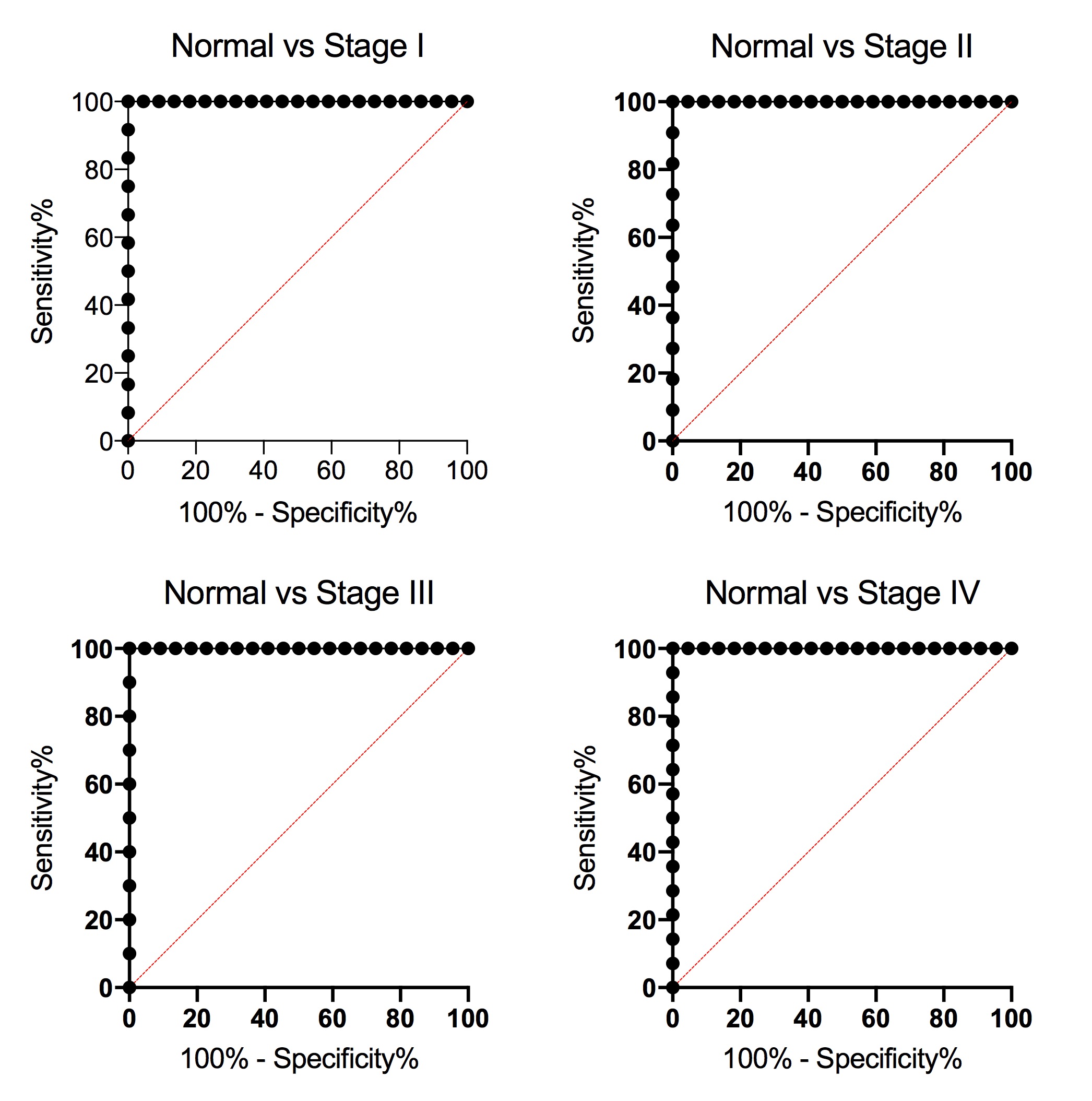

Supplement: Supplementary file 1 — Additional file 1: Supplementary Information. Supplementary Methods. Figure S1. Glycan array analysis of SubB2M and SubBA12 using a Z-Biotech Neu5Ac/Neu5Gc array. A) Glycan array result of SubB2M and SubBA12 performed using the Z-Biotech Neu5Gc/Neu5Ac N-Glycan Array. Histogram represents the average relative fluorescent units of binding to each of the numbered structures shown in B. For structure ID see http://www.zbiotech.com/neu5gc-xenoantigen-microarray.html and http://nebula.wsimg.com/deda6829116ce09edb871bd7ce7cde6c?AccessKeyId=B5CD53DB37409833427C&disposition=0&alloworigin=1 for further information. Figure S2. Characterization of human CA125 O-glycosylation and bovine Alpha-1-acid glycoprotein (bAGP) by PGC-LC-MS/MS. Annotated Base Peak Chromatogram of the total A) O-glycome released from CA125 and Extracted ion chromatogram of m/z 681.32− (Neu5Gc) and 665.32− (Neu5Ac) and B) N-glycome released from bAGP and Extracted ion chromatogram of m/z 1127.42− (Neu5Gc) and 1111.42− (Neu5Ac). Confirmation of C) Neu5Gc (m/z 681.32−) and Neu5Ac (m/z 665.32−) containing O-glycan structures by MS/MS fragmentation and D) Neu5Gc (m/z 1127.42−) and Neu5Ac (m/z 1111.42−) containing glycan structures by MS/MS fragmentation. Figure S3. A representative Glycoprotein Units (GPUs) standard curve. Bovine AGP (MW = 41–43 kDa; ~ 50%/50% Neu5Ac/Neu5Gc; high total sialic acids) and human CA125 (MW = > 200 kDa, 5–10% Neu5Gc; low total sialic acid) were combined at starting concentrations of 15 μg/ml and 15 units/ml, respectively, in 0.5% normal human serum. This glycoprotein mixture was two-fold serially diluted down to 14.65 ng/ml and 0.0146515 units/ml, respectively, in 0.5% normal human serum. The Response Units (RUs) for each concentration of the standard mixture were determined by subtracting binding due to SubBA12 (flow cell 4) from binding due to SubB2M on flow cell 2 and flow cell 3. RUs obtained for the highest concentration standard was considered 100 GPUs. FC2 = flow cell 2; FC3 [file 12885_2022_9428_MOESM1_ESM.zip › Figure S4R2.jpg]

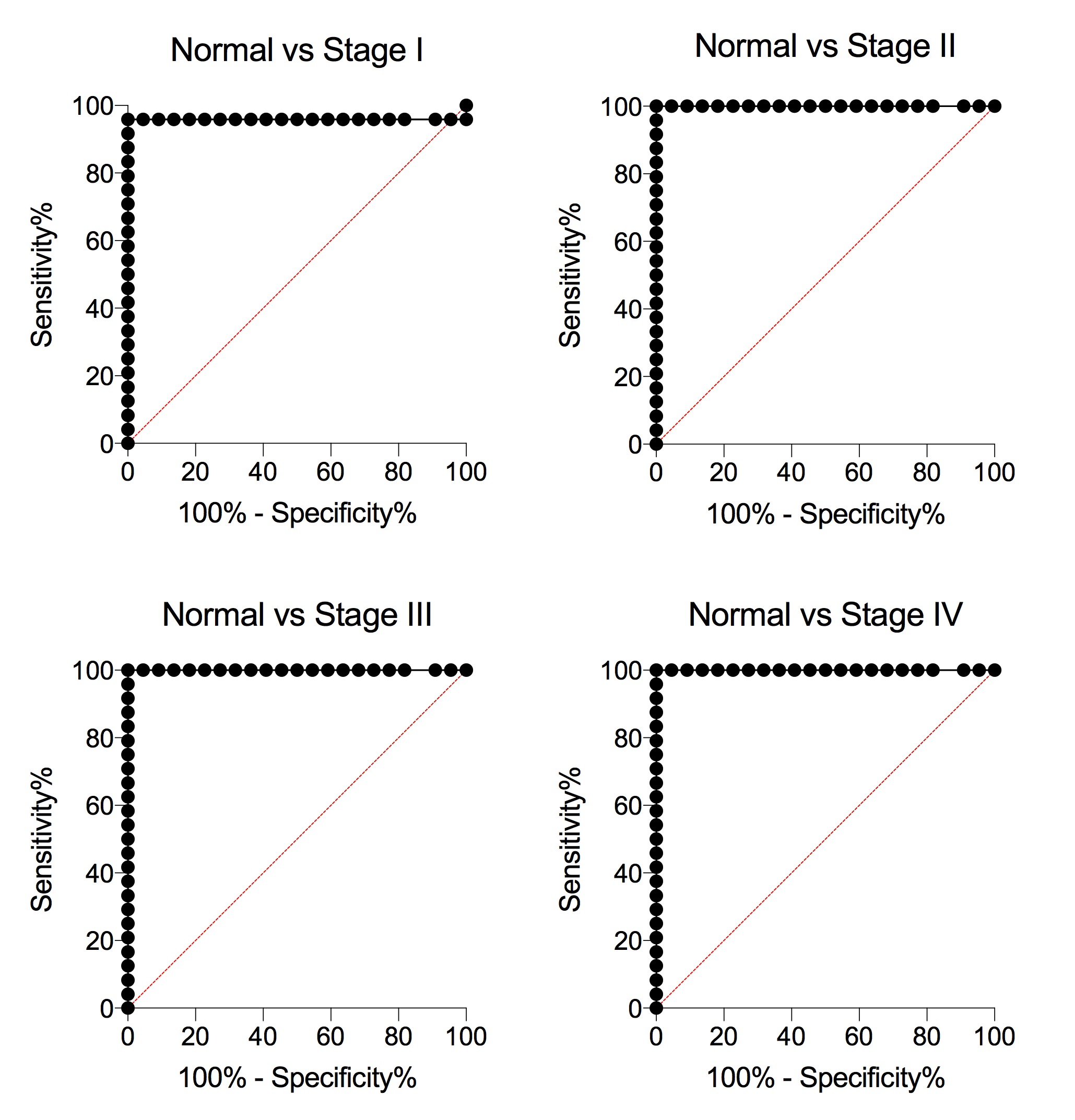

Supplement: Supplementary file 1 — Additional file 1: Supplementary Information. Supplementary Methods. Figure S1. Glycan array analysis of SubB2M and SubBA12 using a Z-Biotech Neu5Ac/Neu5Gc array. A) Glycan array result of SubB2M and SubBA12 performed using the Z-Biotech Neu5Gc/Neu5Ac N-Glycan Array. Histogram represents the average relative fluorescent units of binding to each of the numbered structures shown in B. For structure ID see http://www.zbiotech.com/neu5gc-xenoantigen-microarray.html and http://nebula.wsimg.com/deda6829116ce09edb871bd7ce7cde6c?AccessKeyId=B5CD53DB37409833427C&disposition=0&alloworigin=1 for further information. Figure S2. Characterization of human CA125 O-glycosylation and bovine Alpha-1-acid glycoprotein (bAGP) by PGC-LC-MS/MS. Annotated Base Peak Chromatogram of the total A) O-glycome released from CA125 and Extracted ion chromatogram of m/z 681.32− (Neu5Gc) and 665.32− (Neu5Ac) and B) N-glycome released from bAGP and Extracted ion chromatogram of m/z 1127.42− (Neu5Gc) and 1111.42− (Neu5Ac). Confirmation of C) Neu5Gc (m/z 681.32−) and Neu5Ac (m/z 665.32−) containing O-glycan structures by MS/MS fragmentation and D) Neu5Gc (m/z 1127.42−) and Neu5Ac (m/z 1111.42−) containing glycan structures by MS/MS fragmentation. Figure S3. A representative Glycoprotein Units (GPUs) standard curve. Bovine AGP (MW = 41–43 kDa; ~ 50%/50% Neu5Ac/Neu5Gc; high total sialic acids) and human CA125 (MW = > 200 kDa, 5–10% Neu5Gc; low total sialic acid) were combined at starting concentrations of 15 μg/ml and 15 units/ml, respectively, in 0.5% normal human serum. This glycoprotein mixture was two-fold serially diluted down to 14.65 ng/ml and 0.0146515 units/ml, respectively, in 0.5% normal human serum. The Response Units (RUs) for each concentration of the standard mixture were determined by subtracting binding due to SubBA12 (flow cell 4) from binding due to SubB2M on flow cell 2 and flow cell 3. RUs obtained for the highest concentration standard was considered 100 GPUs. FC2 = flow cell 2; FC3 [file 12885_2022_9428_MOESM1_ESM.zip › Figure S5R2.jpg]

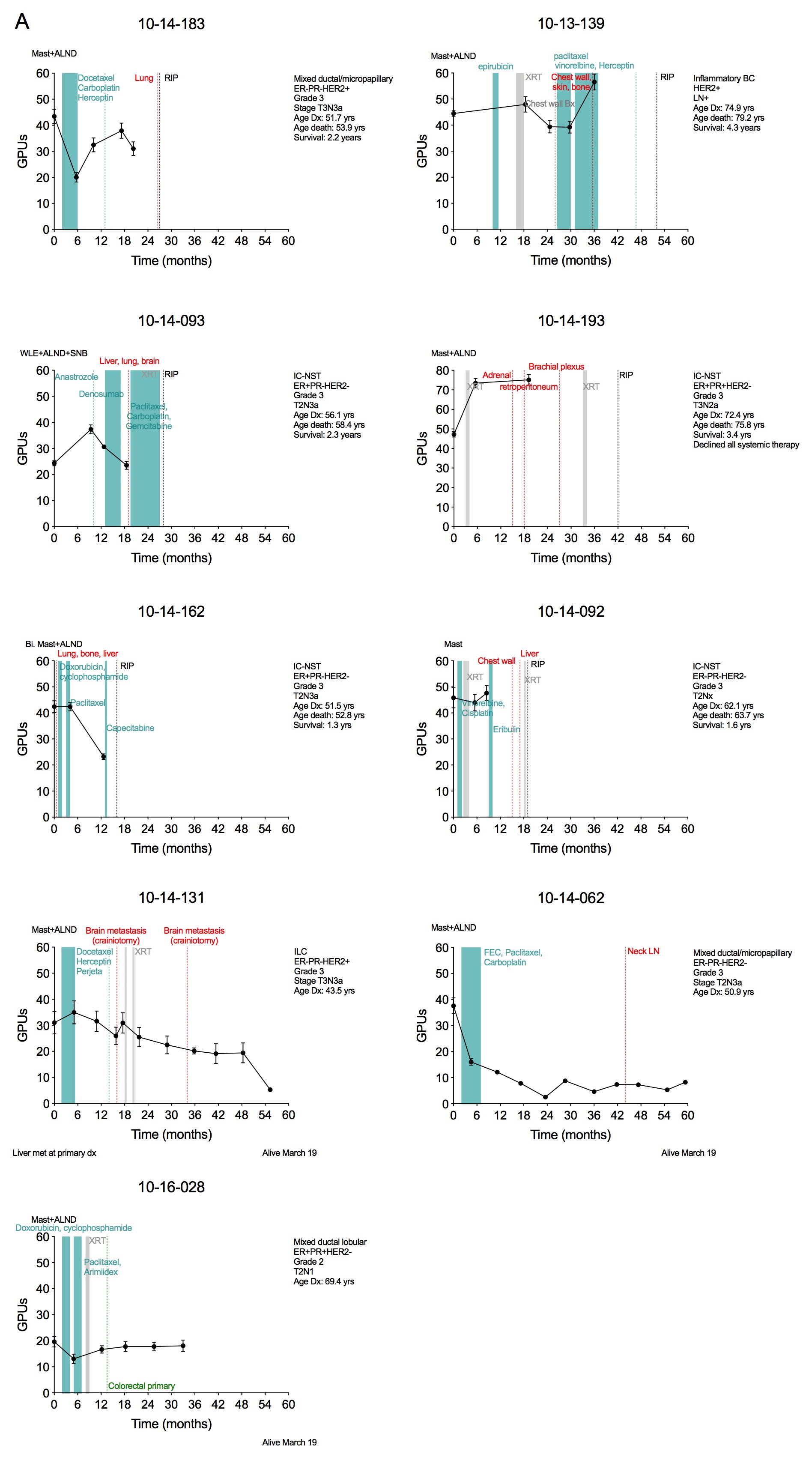

Supplement: Supplementary file 1 — Additional file 1: Supplementary Information. Supplementary Methods. Figure S1. Glycan array analysis of SubB2M and SubBA12 using a Z-Biotech Neu5Ac/Neu5Gc array. A) Glycan array result of SubB2M and SubBA12 performed using the Z-Biotech Neu5Gc/Neu5Ac N-Glycan Array. Histogram represents the average relative fluorescent units of binding to each of the numbered structures shown in B. For structure ID see http://www.zbiotech.com/neu5gc-xenoantigen-microarray.html and http://nebula.wsimg.com/deda6829116ce09edb871bd7ce7cde6c?AccessKeyId=B5CD53DB37409833427C&disposition=0&alloworigin=1 for further information. Figure S2. Characterization of human CA125 O-glycosylation and bovine Alpha-1-acid glycoprotein (bAGP) by PGC-LC-MS/MS. Annotated Base Peak Chromatogram of the total A) O-glycome released from CA125 and Extracted ion chromatogram of m/z 681.32− (Neu5Gc) and 665.32− (Neu5Ac) and B) N-glycome released from bAGP and Extracted ion chromatogram of m/z 1127.42− (Neu5Gc) and 1111.42− (Neu5Ac). Confirmation of C) Neu5Gc (m/z 681.32−) and Neu5Ac (m/z 665.32−) containing O-glycan structures by MS/MS fragmentation and D) Neu5Gc (m/z 1127.42−) and Neu5Ac (m/z 1111.42−) containing glycan structures by MS/MS fragmentation. Figure S3. A representative Glycoprotein Units (GPUs) standard curve. Bovine AGP (MW = 41–43 kDa; ~ 50%/50% Neu5Ac/Neu5Gc; high total sialic acids) and human CA125 (MW = > 200 kDa, 5–10% Neu5Gc; low total sialic acid) were combined at starting concentrations of 15 μg/ml and 15 units/ml, respectively, in 0.5% normal human serum. This glycoprotein mixture was two-fold serially diluted down to 14.65 ng/ml and 0.0146515 units/ml, respectively, in 0.5% normal human serum. The Response Units (RUs) for each concentration of the standard mixture were determined by subtracting binding due to SubBA12 (flow cell 4) from binding due to SubB2M on flow cell 2 and flow cell 3. RUs obtained for the highest concentration standard was considered 100 GPUs. FC2 = flow cell 2; FC3 [file 12885_2022_9428_MOESM1_ESM.zip › Figure S6AR2.jpg]

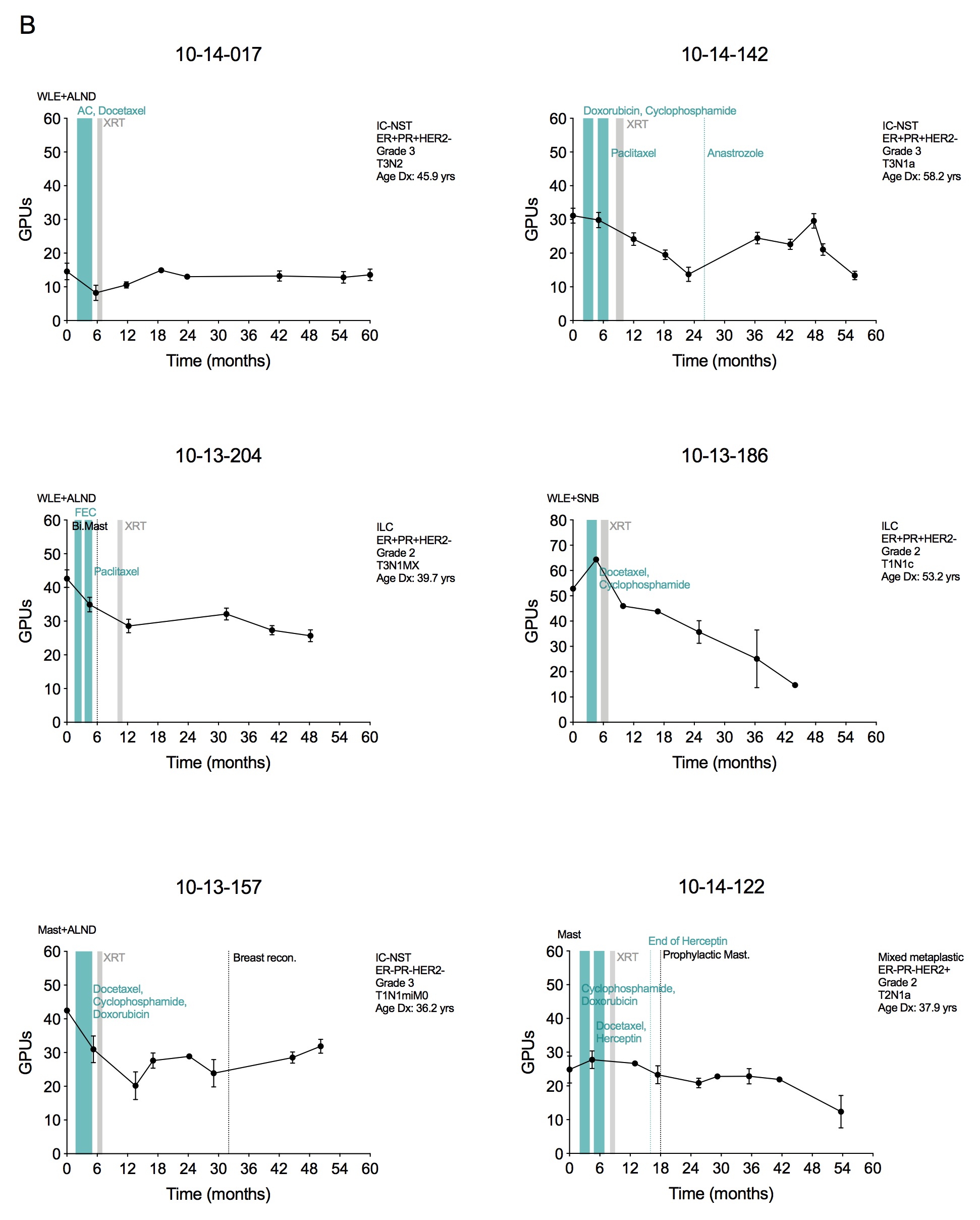

Supplement: Supplementary file 1 — Additional file 1: Supplementary Information. Supplementary Methods. Figure S1. Glycan array analysis of SubB2M and SubBA12 using a Z-Biotech Neu5Ac/Neu5Gc array. A) Glycan array result of SubB2M and SubBA12 performed using the Z-Biotech Neu5Gc/Neu5Ac N-Glycan Array. Histogram represents the average relative fluorescent units of binding to each of the numbered structures shown in B. For structure ID see http://www.zbiotech.com/neu5gc-xenoantigen-microarray.html and http://nebula.wsimg.com/deda6829116ce09edb871bd7ce7cde6c?AccessKeyId=B5CD53DB37409833427C&disposition=0&alloworigin=1 for further information. Figure S2. Characterization of human CA125 O-glycosylation and bovine Alpha-1-acid glycoprotein (bAGP) by PGC-LC-MS/MS. Annotated Base Peak Chromatogram of the total A) O-glycome released from CA125 and Extracted ion chromatogram of m/z 681.32− (Neu5Gc) and 665.32− (Neu5Ac) and B) N-glycome released from bAGP and Extracted ion chromatogram of m/z 1127.42− (Neu5Gc) and 1111.42− (Neu5Ac). Confirmation of C) Neu5Gc (m/z 681.32−) and Neu5Ac (m/z 665.32−) containing O-glycan structures by MS/MS fragmentation and D) Neu5Gc (m/z 1127.42−) and Neu5Ac (m/z 1111.42−) containing glycan structures by MS/MS fragmentation. Figure S3. A representative Glycoprotein Units (GPUs) standard curve. Bovine AGP (MW = 41–43 kDa; ~ 50%/50% Neu5Ac/Neu5Gc; high total sialic acids) and human CA125 (MW = > 200 kDa, 5–10% Neu5Gc; low total sialic acid) were combined at starting concentrations of 15 μg/ml and 15 units/ml, respectively, in 0.5% normal human serum. This glycoprotein mixture was two-fold serially diluted down to 14.65 ng/ml and 0.0146515 units/ml, respectively, in 0.5% normal human serum. The Response Units (RUs) for each concentration of the standard mixture were determined by subtracting binding due to SubBA12 (flow cell 4) from binding due to SubB2M on flow cell 2 and flow cell 3. RUs obtained for the highest concentration standard was considered 100 GPUs. FC2 = flow cell 2; FC3 [file 12885_2022_9428_MOESM1_ESM.zip › Figure S6BR2.jpg]
